# Right extrapleural hematoma due to thoracic trauma. The extrapleural fat sign

**DOI:** 10.36416/1806-3756/e20250027

**Published:** 2025-03-18

**Authors:** Raquel García-Latorre, Luis Gorospe, Abel González-Huete

**Affiliations:** 1. Servicio de Radiología, Hospital Universitario Ramón y Cajal, Madrid, España.

We report the case of a 54-year-old male presenting to the emergency department with dyspnea and right-sided chest pain following recent thoracic trauma. He had no significant medical history but showed progressive anemia.

A chest X-ray (Figure 1A) revealed right-sided rib fractures and an extensive ipsilateral opacity with extrapulmonary morphology. Chest CT scan (Figures 1B-D) showed a loculated, biconvex collection on the right side, with dependent areas of bleeding, separated from the lung parenchyma by a thin fat-density line (the extrapleural fat sign). These findings confirmed an extrapleural hematoma. The hematoma was successfully drained via a chest tube, leading to clinical improvement.

**Figure 1:**
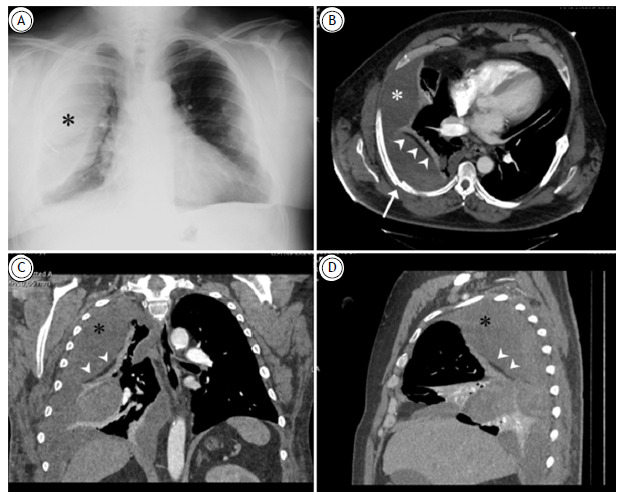
(A) Posteroanterior chest X-ray showing right rib fractures and a large extrapulmonary opacity (asterisk). Axial (B), coronal (C), and sagittal (D) reconstructions of chest CT scan (mediastinal window) reveal rib fractures (arrow) and a large extrapulmonary collection (asterisks) separated from the atelectatic lung parenchyma by a linear fat-density image (arrowheads) corresponding to the extrapleural fat sign, consistent with an extrapleural hematoma.

Extrapleural hematomas are rare, occurring in 7.1% of thoracic trauma cases. They result from bleeding between the parietal pleura and endothoracic fascia and are often associated with rib fractures, hemothorax, pneumothorax, and pulmonary contusions.^1^

The extrapleural fat sign, seen on CT, is a linear fat-density line separating the pulmonary parenchyma from extrapleural lesions. It corresponds to extrapleural fat thickened and medially displaced in extrapleural pathologies.^2^

Recognizing this sign is critical to differentiating extrapleural hematomas from hemothorax, as their management and complications differ.^1^ ^,^ ^2^

## References

[1] Shankar T, Ameena Ms S, Nagasubramanyam V, Meena R, Sasidharan P (2024). Extrapleural Hematoma A Rare Sequalae of Thoracic Trauma. Cureus.

[2] Chung JH, Carr RB, Stern EJ (2011). Extrapleural hematomas imaging appearance, classification, and clinical significance. J Thorac Imaging.

